# Sirtuin1, not NAMPT, possesses anti-inflammatory effects in epicardial, pericardial and subcutaneous adipose tissue in patients with CHD

**DOI:** 10.1186/s12967-023-04518-4

**Published:** 2023-09-21

**Authors:** Trine Baur Opstad, Bianca Papotti, Sissel Åkra, Charlotte Holst Hansen, Bjørn Braathen, Theis Tønnessen, Svein Solheim, Ingebjørg Seljeflot

**Affiliations:** 1 Center for Clinical Heart Research, Department of Cardiology, Oslo University, Hospital Ullevål, Pb 4954 Nydalen, 240 Oslo, Norway; 2 Faculty of Medicine, University of Oslo, Oslo, Norway; 3 Department of Food and Drug, University of Parma, Parma, Italy; 4 Department of Cardiothoracic Surgery, Oslo University Hospital, Oslo, Norway

**Keywords:** SIRT1, NAMPT, Cardiac adipose tissue, Inflammation, CHD

## Abstract

**Background:**

Inflammation in cardiac adipose tissue (AT) is associated with atherosclerosis. We investigated whether the epicardial-, pericardial and pre-sternal subcutaneous AT (EAT, PAT and SAT) expression of Sirtuin1 (SIRT1) and nicotinamide phosphoribosyl transferase (NAMPT) are involved in the inflammatory process in coronary heart disease (CHD), and potentially associated to nod-like receptor family pyrin domain containing 3 (NLRP3) inflammasome-related markers, macrophage polarization markers, cell markers and the cardiometabolic profile.

**Methods:**

In this cohort study performed between 2016 and 2018, EAT, PAT and SAT biopsies were retrieved from 52 CHD patients (77% men, median age 67) undergoing open-chest coronary artery bypass grafting (CABG), and 22 patients (50% men, median age 69) undergoing aortic valve replacement serving as controls. AT samples were snap-frozen at – 80 °C until RNA extraction and AT expression of actual markers, relatively quantified by PCR. Circulating SIRT1 and NAMPT were measured with Enzyme-linked immunosorbent assays (ELISAs). Non-parametric statistical tests were mainly used, including Friedman’s test coupled to Wilcoxon signed-rank test and Spearman Correlation.

**Results:**

SIRT1 and NAMPT levels were similar in CHD and controls. In CHD, SIRT1 and NAMPT were inter-correlated in all AT compartments (r = 0.37–0.56, *p* < *0.01*, all), and differently expressed between compartments, with the highest expression in SAT, significantly different from EAT (*p* < *0.01*, both). Circulating SIRT1 and NAMPT levels were inversely associated (r = − 0.32, *p* = *0.024*). In EAT and SAT, SIRT1 expression was inversely associated with IL-18 (r = − 0.43 and r = − 0.38, *p* < *0.01*, both), whereas NAMPT expression was positively associated with the NLRP3 inflammasome-related markers in all compartments (r = 0.37–0.55, *p* < *0.01*, all). While SIRT1 and NAMPT correlated to nitric oxide synthase 2 (NOS2), especially in SAT (r = 0.50–0.52, *p* ≤ *0.01*, both), SIRT1 expression was related to endothelial cells, and NAMPT to macrophages. SIRT1 levels were correlated to weight and waist (r = 0.32 and r = 0.38, *p* < *0.03*, both) and inversely to triglycerides and glycated haemoglobin (HbA1c) (r = − 0.33–− 0.37, *p* < *0.03*, all), the latter positively correlated to NAMPT concentration (r = 0.39, *p* = *0.010*).

**Conclusion:**

The study indicates that targeting SIRT1, with its anti-inflammatory properties, may be a novel anti-inflammatory strategy in preventing atherosclerosis and CHD progression. NAMPT may be an early player in AT inflammation, mediating/reflecting a pro-inflammatory state.

*Trial Registration*: *Registration: Clinicaltrials.gov ID: NCT02760914, registered the 5th of February 2016, *http://clinicaltrials.gov/NCT02760914

**Graphical Abstract:**

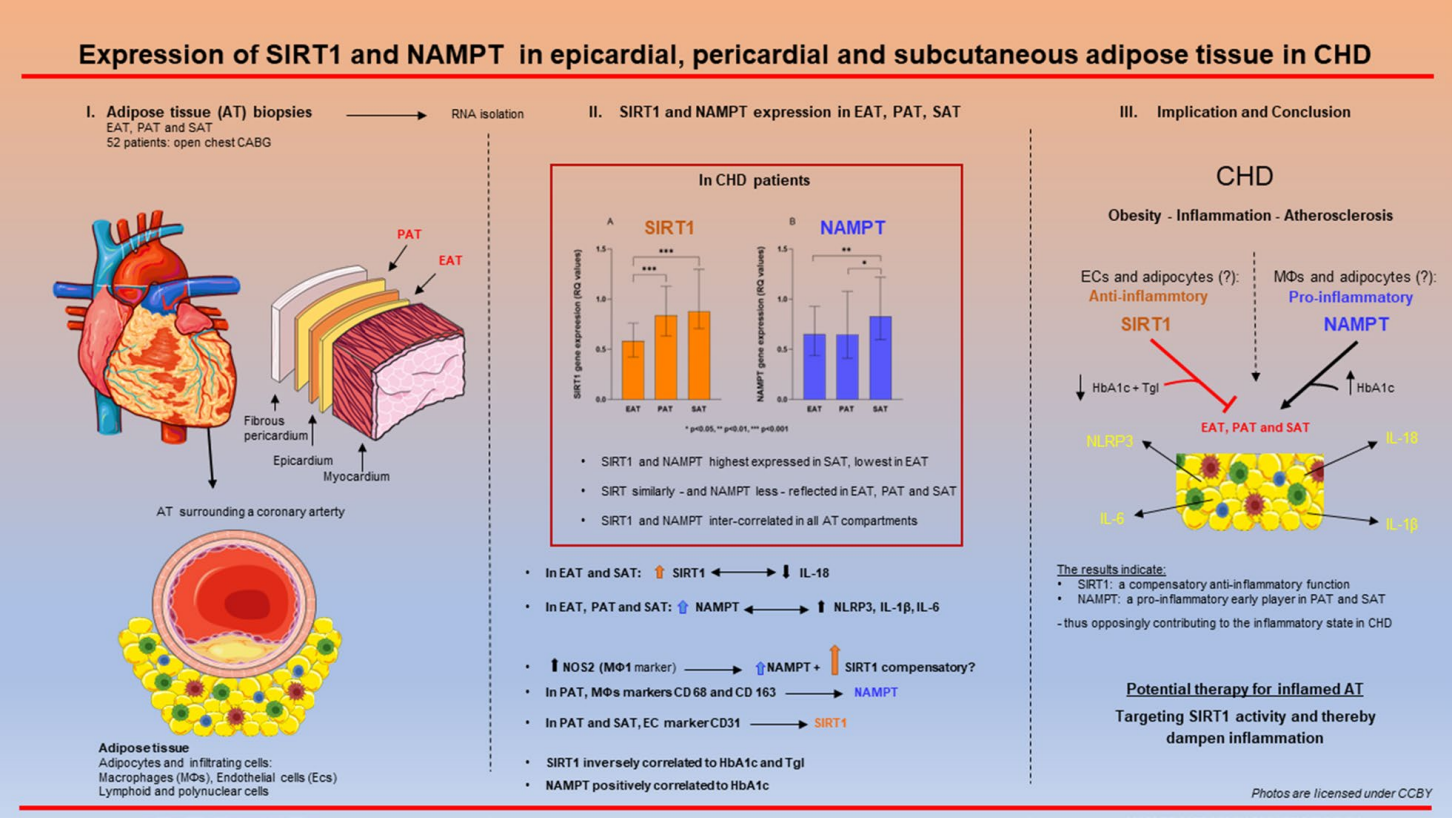

**Supplementary Information:**

The online version contains supplementary material available at 10.1186/s12967-023-04518-4.

## Introduction

Ageing, metabolic disorders, obesity and chronic low-grade inflammation are contributors to the pathogenesis of atherosclerosis. The pathophysiology of vascular aging involves oxidative stress as an early stage in its development [1]. In obesity, when the subcutaneous adipose tissue (AT) has exceeded its capacity to expand, it becomes dysfunctional and fat starts to accumulate in ectopic tissue, including the heart, with an increase in epicardial AT (EAT) and pericardial or paracardial AT (PAT) volume [2]. EAT, located directly adjacent to the myocardium and surrounding the coronary arteries, shares blood supply with the myocardium [3]. PAT, located between the visceral and parietal pericardium, shares microcirculation with non-coronary arteries. As such, the specific release of inflammatory signals from EAT and PAT may, based also on their diverse origin, have distinct effects, and reflect different inflammatory profiles [4]. Whether subcutaneous AT (SAT) reflects inflammatory activity in EAT and PAT, is not clear. We have shown that expression of nod-like receptor family pyrin domain containing 3 (NLRP3) inflammasome-related cytokines, to some degree, were mirrored in EAT, PAT and SAT [5]. A dysregulated or inflamed AT, often seen in obesity, is infiltrated by pro-inflammatory macrophages (M1mɸ) and other immune cells, resulting in a local production of cytokines [6, 7]. If EAT is affected, this could directly affect the myocardium due to its anatomical proximity, potentially changing contractility of the cardiomyocytes and eventually also accelerating the atherosclerotic process in the coronary arteries [8, 9]. The anti-inflammatory macrophages (M2mɸ) seem to dominate in lean subjects, although less extensively [7].

SIRT1 may be a promising new target in the treatment of cardiovascular disease, with its potential to prevent cell aging [10]. SIRT1 is a nicotinamide adenine dinucleotide (NAD)+ deacetylase mainly located in the nucleus, of which targets are histones, and non-histones proteins including several transcription factors involved in regulation of metabolic processes and in inhibition of oxidative stress, inflammation, and apoptosis [11]. The pleiotropic effects of SIRT1 seem also to include Mɸ polarization, promoting M2mɸ differentiation [12]. SIRT1 was recently reported to inhibit hypoxia-induced oxidative stress in cardiomyocytes [13] and its circulating levels in obese were further reported to be inversely associated to epicardial fat thickness [14]. We did recently show that an elevation of SIRT1 concentration after four years of selenium and coenzyme Q_10_ intervention associated with reduced cardiovascular mortality potentially through anti-oxidative mechanisms [15].

The activity of SIRT1 is partly based on the availability of its coenzyme NAD+ , which is biosynthesized from nicotinamide, by nicotinamide phosphoribosyl transferase (NAMPT) [16], also named Visfatin. NAMPT is preferentially produced by the visceral adipose tissue [17], although also demonstrated in SAT [18], in immune cells and cardiomyocytes, among others [19, 20]. Intracellular NAMPT acts as a rate-limiting enzyme in NAD biosynthesis. Extracellular NAMPT also is involved in NAD formation, and/or acts as a cytokine possessing both pro-and anti-inflammatory properties, possibly mainly the first, and with the ability to mediate Mɸ1 differentiation [21, 22] Increased circulating NAMPT levels have been reported in subjects with metabolic dysfunction such as in diabetes type 2 with additionally advanced carotid atherosclerosis [23], and was also associated with atherogenic inflammatory markers and endothelial dysfunction [24]. In contrast, we could show that NAMPT expression in SAT, retrieved from the gluteal region, was inversely associated with the amount of abdominal SAT and with Insulin and C-peptide in healthy middle-aged men [25].

Here, we aimed to explore the profile of SIRT1 and NAMPT expression in EAT, PAT and SAT in patients with CHD and individuals with aortic valvular disease. Any association with inflammatory- and Mɸ polarization markers, specific cell markers and cardiometabolic variables was assessed. Our hypothesis was that SIRT1 and NAMPT might be differentially expressed in EAT and PAT, partly reflected in SAT, with associations to anti- and proinflammatory processes, respectively.

## Methods

The data that support the findings of this study are available from the corresponding author upon reasonable request. Study subjects included patients with CHD undergoing open-chest coronary artery bypass grafting (CABG) (n = 52), and subjects eligible for aortic valve replacement without evidence of coronary heart disease (CHD) as controls (n = 22), recruited at Oslo University Hospital, Ullevål in Norway in the period from December 2016 to May 2018. Clinical characteristics were registered the day before surgery. No restrictions were set for inclusion, other than exclusion of subjects on steroid drugs.

During the open-chest surgery and before starting the extracorporeal circulation, biopsies from EAT, PAT and SAT were retrieved, processed, and snap-frozen at – 80 °C until RNA extraction and gene-expression analysis. EAT was taken between the right coronary artery and the pulmonary artery, PAT ventrally to the pericardium in front of aorta, and SAT was taken pre-sternally at the middle of sternum.

### Laboratory analyses

Arterial blood samples were collected from each patient at the start of anaesthesia. Whole blood was centrifuged at 2500 g for 10′, and serum samples were kept frozen at – 80 °C until the analysis of SIRT1 and NAMPT, using the Human SIRT1 and Visfatin (NAMPT) Enzyme-linked immunosorbent assay (ELISA) kits from LSBio LifeSpan BioSciences (Seattle, USA) and MyBioSource (California, USA), respectively. SIRT1 and NAMPT were successfully measured in 72 samples, respectively, with an inter-assay coefficient of variation of 13.5% and 7.4, respectively. Routine analyses were performed by conventional laboratory methods.

### RNA isolation and SIRT1 and NAMPT gene expression

Total RNA was extracted from the AT samples by using the RNeasy Lipid Tissue Mini Kit (Qiagen, GmbH, Germany) following instructions from the manufacturer. RNA quantity and purity were determined with the Nanodrop device capable of spectrophotometric measurements (Thermo Scientific NanoDrop^™^, USA), giving a mean concentration in the samples of 28.6 ng/mL and a purity of 1.7 (ratio of assessed absorbance at 260 and 280 nm). cDNA was retro-transcribed from equal amounts of total RNA (5 ng/mL) using the qScript^™^ cDNA superMix (Quanta Biosciences, Maryland, USA). Gene expression of SIRT1 (n = 72) and NAMPT (n = 71) were measured using the TaqMan^®^ assays Hs01009006_m1 and Hs00237184_m1, respectively, and the TaqMan^®^ Universal PCR Master Mix (P/N 4324018,) on the ViiA 7 instrument (all Applied Biosystems, CA, USA). The β2-microglobulin (Hs99999907_m1, Applied Biosystems) was used as the normalizer internal gene, and relative quantification of SIRT1 mRNA and NAMPT mRNA levels were determined applying the ΔΔCt method [26]. Gene expression of the NLRP3-related inflammatory markers (NLRP3, Caspase-1, IL-18, IL-1β, the macrophage polarization markers nitric oxide synthase 2 (NOS2) (Mɸ1) and scavenging mannose receptor CD206 (Mɸ2), and the cell markers cluster of differentiation** (**CD) 3 (T-cells), CD31 (endothelial cells), CD68 and CD163 (macrophages), were previously analysed as indicated [27, 28].

### Statistics

Clinical characteristics are reported as numbers and percentages, or as median values with 25th and 75th percentiles. As SIRT1 and NAMPT levels were mostly skewed distributed, non-parametric tests were used throughout. The Friedman’s test coupled to Wilcoxon signed-rank test was used to compare SIRT1 and NAMPT gene expression between the different AT compartments. Spearman Rho was used for the correlation analyses, with correction for multiple testing by Bonferroni. A *p-value* < *0.05* was considered statistically significant. SPSS version 27 was used throughout (SPSS lnc. ILL, USA).

## Results

Clinical characteristics of the study population are summarized in Table1, divided according to patients having CHD allocated to CABG, and subjects without CHD eligible for valve replacement (Controls) (Placed at the end of text, page 25–26). The presence of men and comorbidity was higher in the CHD group, as was also the use of statins and beta-blockers, along with higher HbA1c levels and glomerular filtration rate compared to the control group. Total Cholesterol and LDL-C were lower in CHD patients probably due to the higher frequency of lipid lowering drugs.

**Table 1:** Clinical characteristics in the CHD patients and Controls *(page 8, l. 204)*

	CHD patients	Controls	p value
Age (years)	66.5 (62,71.8)	69 (63, 71.5)	
Male (%)	40 (76.9%)	11 (50%)	0.04
Smoker (previous/current)	31 (59.6%)	10 (45.5%)	
Weight (Kg)	85 (70.3, 95.5)	82.5 (77.5, 107.0)	
Height (m)	1.77 (1.68, 1.81)	1.75 (1.67, 1.82)	
Waist (cm)	92 (86, 98)	90 (88, 101)	
BMI (kg/m^2^)	27.3 (23.8, 30.1)	28.4 (24.6, 31.6)	
SBP (mmHg)	140 (125, 160)	140 (115, 163)	
DBP (mmHg)	80 (70, 87)	79 (70, 86)	
Cardiovascular status			
Previous AMI (%)	20 (38.5%)	2 (9.1%)	0.025
Angina (%)	24 (46.2%)	0 (0%)	< 0.001
PCI (%)	20 (38.5%)	0 (0%)	0.002
Hypertension (%)	28 (53.9%)	9 (40.9%)	
Diabetes type I and II (%)	14 (26.9%)	3 (13.6%)	
Heart failure (%)	3 (5.8%)	1 (4.5%)	
Medications			
Aspirin (%)	45 (86.5%)	9 (40.9%)	< 0.001
Other antiplatelet (%)	14 (26.9%)	0 (0%)	
ACEi/ATII (%)	24 (46.2%)	11 (50%)	
Beta-blockers (%)	32 (61.5%)	6 (27.3%)	0.015
Statins (%)	37 (71.2%)	11 (50%)	
Other lipid-lowering agents (%)	10 (19.2%)	1 (4.5%)	
Insulin (%)	6 (11.5%)	0 (0%)	
Anti-diabetic drugs (%)	11 (21.2%)	3 (13.6%)	
Diuretics (%)	7 (13.5%)	5 (22.7%)	
Laboratory values			
hsCRP (mg/L)	0.91 (0.49, 1.77)	1.00 (1.00, 2.00)	
Troponin T (ng/L)	13 (9, 22)	11.5 (9, 25)	
TC (mmol/L)	3.1 (2.7, 3.4)	3.9 (2.8, 4.6)	0.026
HDL-C (mmol/L)	0.97 (0.75, 1.12)	1.10 (0.87, 1.30)	
LDL-C (mmol/L)	1.83 (1.4, 2.2)	2.18 (1.8, 2.9)	0.02
Triglycerides (mmol/L)	1.22 (1.0, 1.8)	1.02 (0.9, 1.7)	
Glucose (mmol/L)	5.6 (4.9, 6.6)	5.6 (4.9, 6.3)	
HbA1c (mmol/mol)	39 (36, 51)	36 (33, 38)	0.011
GFR (%)	90 (75, 95)	80 (68, 91)	0.03
Creatinine (μmol/L)	76 (67, 85)	80 (64.8, 91)	
Uric acid (μmol/L)	313 (271, 362)	317 (258, 396)	

Levels are median (25, 75 percentiles) or number (% frequency). P-values < 0.05 are shown

*BMI* Body Mass Index, *SBP* Systolic Blood Pressure, *DBP* Diastolic Blood Pressure, *AMI* Acute Myocardial Infarction, *PCI* Percutaneous Coronary Intervention, *ACEi* Angiotensin-Converting Enzyme inhibitors, *ATII* Angiotensin II receptor antagonist, *NSAIDs* non-steroidal anti-inflammatory drugs, *hsCRP* high sensitive C-reactive protein, *TC* total cholesterol, *HDL-C* high-density lipoprotein cholesterol, *LDL-C* low-density lipoprotein cholesterol, *HbA1C* glycosylated haemoglobin A1c, *GFR* Glomerular filtration rate

As no imaging studies were performed to verify that the control group were indeed free of CHD and the observation that SIRT1 and NAMPT AT expression and circulating levels did not differ significantly between CHD and controls (Table 2), further results will exclusively be presented in CHD patients.

**Table 2:** Expression and circulating levels of SIRT1 and NAMPT in CHD patients and Controls

	CHD patients	Controls	*p-value*
SIRT1 expression EAT	0.583 (0.425, 0.761)	0.529 (0.408, 0.744)	*0.75*
PAT	0.838 (0.636, 1.129)	0.891 (0.708, 1.129)	*0.30*
SAT	0.879 (0.707, 1.298)	1.143 (0.935, 1.482)	*0.12*
Circulating SIRT1 ng/mL	181.9 (103.8, 316.8)	143.3 (99.0, 225.6)	*0.29*
NAMPT expression EAT	0.653 (0.437, 0.928)	0.793 (0.434, 1.034)	*0.44*
PAT	0.646 (0.410, 1.078)	0.576 (0.425, 0.884)	*0.76*
SAT	0.827 (0.599, 1.219)	0.911 (0.727, 1.041)	*0.83*
Circulating NAMPT ng/mL	1.597 (1.499, 1.815)	1.630 (1.482, 1.781)	*0.95*

Levels are median values (25, 75 percentiles)

EAT, PAT, SAT; epicardial-, pericardial-, subcutaneous adipose tissue

### SIRT1 and NAMPT expression in EAT, PAT and SAT in CHD

**Fig. 1:**
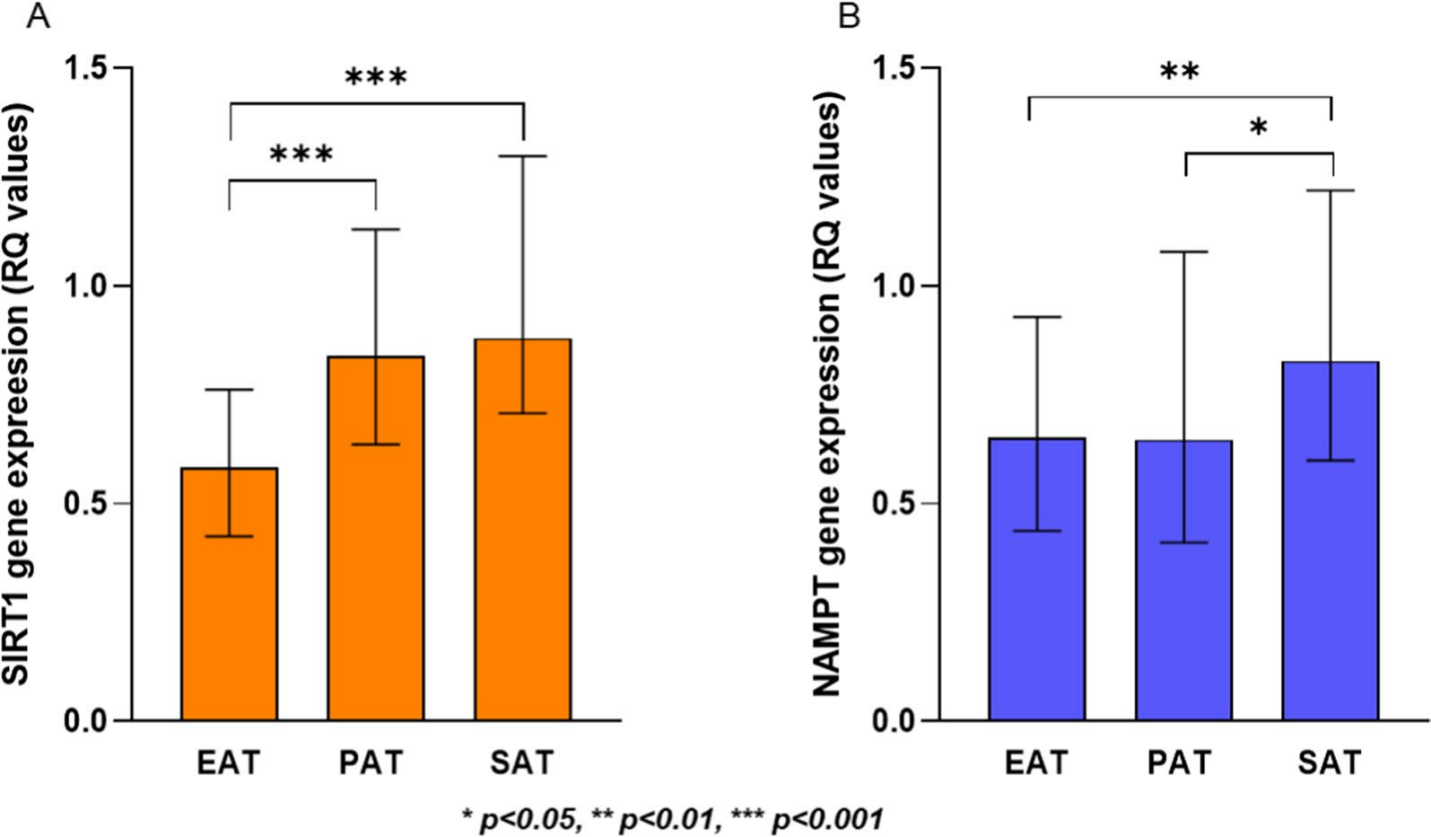
Relatively quantified (RQ) expression of SIRT1 (A) and NAMPT (B) in AT compartments. **A** SIRT1 gene expression in EAT, PAT and SAT (orange columns) in CHD patients (n = 52). A *p-value* < *0.001* is specified with ***, for the difference in SIRT1 expression between EAT and SAT and EAT and PAT, respectively. The difference between PAT and SAT was not statistically significant (*p* = *0.62*). The Friedman’s test coupled to Wilcoxon signed-rank test was used. **B** NAMPT gene expression in EAT, PAT and SAT (blue columns) in CHD patients (n = 50). A *p-value of 0.003* for the difference in NAMPT expression between EAT and SAT is specified with **, and a *p-value of 0.016* is specified with * for the difference between PAT and SAT. The difference between EAT and PAT was not statistically significant (*p* = *0.55*). The Friedman’s test coupled to Wilcoxon signed-rank test was used

SIRT1 was differently expressed in the three AT compartments. As shown in Fig. 1 A, the lowest expression was observed in EAT, and significantly lower than in SAT and PAT, respectively (*p* < *0.001,* both), whereas the expression in SAT was not significantly different from that in PAT (*p* > *0.40*).

NAMPT was also differently expressed in EAT, PAT and SAT (*p* = *0.006*), with the highest levels in SAT compared to both EAT and PAT (*p* = *0.003 and p* = *0.016*, respectively), and with no significant difference between EAT and PAT (Fig. 1B).

### Intra- and inter correlations of SIRT1 and NAMPT expression between different AT compartments and their respective circulating levels in CHD

Significant correlations of SIRT1 expression were observed between EAT, PAT and SAT, and SIRT1 expression in SAT correlated to SIRT1 circulating levels (Table 3). For NAMPT, weaker but significant correlations were observed between the AT compartments (Table 3). After correction for multiple testing (six performed correlations, giving a cut-off value for significance of *p* = *0.008*), the correlations between SIRT1 in SAT, EAT and PAT, respectively, remained statistically significant.

**Table 3:** Intra-correlations of SIRT1 and NAMPT between different AT compartments at their circulating levels in CHD

	SIRT1 PAT	SIRT1 SAT	SIRT1 ng/mL
SIRT1 EAT	**r = 0.355, *p* = *0.015***	r = 0.455, *p* = 0.001*	r = 0.218, *p* = *0.12*
SIRT1 PAT		r = 0.530, *p* < 0.001*	r = 0.133, *p* = *043*
SIRT1 SAT			**r = 0.305, *p* = *0.029***

	NAMPT PAT	NAMPT SAT	NAMPT ng/mL
NAMPT EAT	**r = 0.329, *p* = *0.017***	**r = 0.285, *p* = *0.045***	r = -0.023, *p* = *0.87*
NAMPT PAT		**r = 0.297, *p* = *0.036***	r = -0.093, *p* = *0.52*
NAMPT SAT			r = 0.021, *p* = *0.89*

Bolded text indicates statistically significant correlations

^*^Statistically significant after correcting for multiple testing

Expression of SIRT1 and NAMPT inter-correlated significantly in EAT, PAT and SAT (*p* < *0.01*, all) and SIRT1 in PAT also correlated significantly to NAMPT in SAT (*p* = *0.001*) (Table 4). Circulating SIRT1 levels were inversely associated with circulating NAMPT. After correction for multiple testing (16 performed correlations giving a cut-off value for significance at *p* = *0.003*), the markers inter-correlation in SAT and in the circulation were no longer statistically significant.

**Table 4:** Inter-correlations between SIRT1 and NAMPT in different AT compartments at their circulating levels in CHD

	NAMPT EAT	NAMPT PAT	NAMPT SAT	NAMPT ng/mL
SIRT1 EAT	r = 0.560, *p* < 0.001*	r = 0.174, *p* = *0.22*	r = 0.241, *p* = *0.092*	r = -0.199, *p* = *0.41*
SIRT1 PAT	r = 0,195, *p* = *0.17*	r = 0.481, *p* < 0.001*	r = 0.0.456, *p* = 0.001*	r = -0.020, *p* = *0.89*
SIRT1 SAT	r = 0.222, *p* = *0.12*	r = 0,248, *p* = *0.080*	**r = 0.0.368, *p* = *0.009***	r = -0.044, *p* = *0.77*
SIRT1 ng/mL	r = 0.256, *p* = *0.067*	r = 0.055, *p* = *0.70*	r = 0.154, *p* = *0.29*	**r = − 0.319, *p* = *0.024***

Bolded text indicates statistically significant correlations

^*^ Statistically significant after correcting for multiple testing

### Correlations of SIRT1 and NAMPT to NLRP3 inflammasome-related markers in CHD

**Fig. 2:**
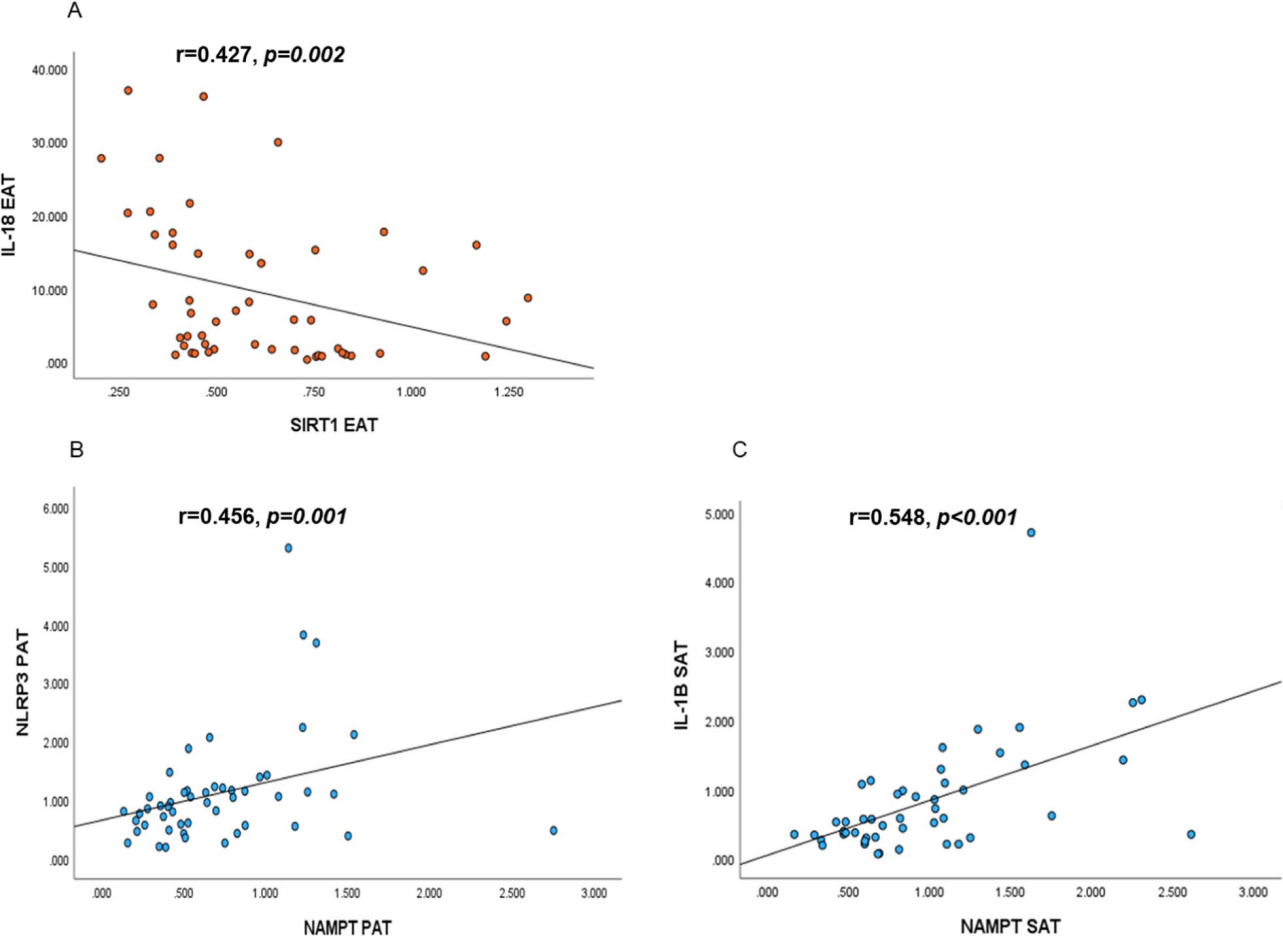
Adjusted significant correlations of SIRT1 and NAMPT to inflammatory markers in different AT compartments. **A** Scatterplot visualizing the inverse correlation between SIRT1 and IL-18 in EAT in CHD patients (n = 51), Spearman Rho = 0.457, *p* = *0.002*. **B** Scatterplot visualizing the correlation between NAMPT and NLRP3 in PAT in CHD patients (n = 51), Spearman Rho = 0.4456, *p* = *0.001.* **C** Scatterplot visualizing the correlation between NAMPT and IL-1β in SAT in CHD patients (n = 48), Spearman Rho = 0.548, *p* < *0.001*

Correlations between the expression of SIRT1 and NAMPT to NLRP3, IL-18, IL-1β, IL-6 and IL-6 receptor (IL-6R) in EAT, PAT and SAT are shown in Additional file 1: Table S1. For SIRT1, the strongest and inverse correlations were found between SIRT1 and IL-18 in EAT (r = − 0.427, *p* = *0.002*) and in SAT (r = − 0.378, *p* = *0.007*). For NAMPT, strong positive correlations were observed between NAMPT and NLRP3 in both EAT and PAT, and between NAMPT and IL-6 in EAT and SAT (r = 0.367 and r = 0.371, *p* < *0.01*, both), and between NAMPT and IL-1β in SAT (r = 0.55, *p* < *0.001*). Results that were still significant after Bonferroni correction (15 performed correlations giving a cut-off value for significance of *p-values* ≤ *0.003*) are shown in Fig. 2A, B, and C.

### Correlations of SIRT1 and NAMPT to NOS2 (Mɸ1) and CD206 (Mɸ2) in CHD

**Fig. 3:**
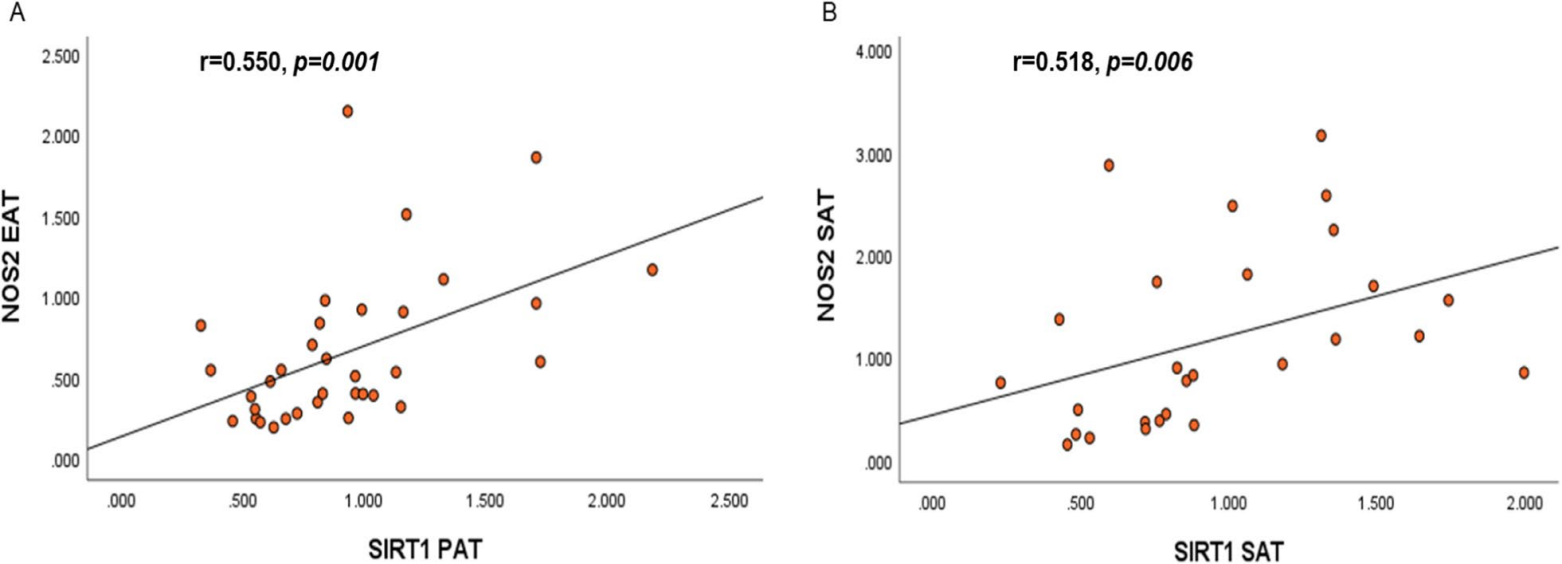
Adjusted significant correlations between SIRT1 and NOS2 in different AT compartments. **A** Scatterplot visualizing the correlation between SIRT1 in PAT and NOS2 in EAT (n = 34), Spearman Rho = 0.550, *p* = *0.001*. **B** Scatterplot visualizing the correlation between SIRT1 and NOS2 in SAT (n = 27), Spearman Rho = 0.518, *p* = *0.006*

Additional file 1: Table S2 shows the correlations between SIRT1 and NAMPT expression and the macrophage polarizations markers CD206 and NOS2 in EAT, PAT and SAT, respectively. SIRT expression in PAT and SAT was significantly correlated to NOS2 expression in all 3 AT compartments, with the strongest correlation between SIRT1 and NOS2 in PAT (r = 448, *p* = *0.011*), between SIRT1 in PAT and NOS2 in EAT (r = 0.550, *p* = *0.001*) and SIRT1 and NOS21 in SAT (r = 0.518, *p* = *0.006*), the two latter still significant after correcting for multiple testing (6 performed correlations between SIRT1, CD206 and NOS2 in each AT compartment giving a cut-off value for significance of; *p* = *0.008*) (Fig. 3A, B). Expression of NAMPT and CD206 were significantly inter-correlated in EAT, as was also NAMPT and NOS2 in SAT and NAMPT expression in PAT correlated to NOS2 in EAT, however, none of these correlations were significant after Bonferroni correction.

### Correlations of SIRT1 and NAMPT to selected cell markers in CHD

To explore potential sources of SIRT1 and NAMPT in the retrieved AT samples, their expression was correlated to selected cell markers. In PAT and SAT, SIRT1 correlated to the endothelial cell marker CD31 (r = 0.277, *p* = *0.049* and r = 0.453, *p* < *0.001*), whereas no significant correlations were observed to the other cell markers CD3 (T-cells), CD68 and CD163 (macrophages). NAMPT correlated significantly to CD68 (r = 0.305, *p* = *0.028*) and inversely to CD3 (r = − 0.313, *p* = *0.024*) in PAT, otherwise no other significant correlations were observed.

### SIRT1 and NAMPTs’ relation to metabolic variables in CHD

As shown in Table 5, circulating SIRT1 levels were significantly correlated to weight and waist (r = 0.384 and r = 0.324, *p* < *0.05*, both), whereas no significant correlations were observed between expression of SIRT1 in any of the compartments and these anthropometric variables. SIRT1 expressed in EAT and SIRT1 circulating levels were inversely correlated to glycated haemoglobin (HbA1c) (r = − 0.371 and r = − 0.350, *p* < *0.02*, both) and SIRT1 expressed in SAT correlated inversely to triglycerides (r = − 0.327, *p* = *0.022*). For NAMPT, the only significant correlation was between its circulating levels and HbA1c (r = 0.386, *p* = *0.010*). After Bonferroni correction (24 performed correlations giving a cut-off value for significance of; *p* = *0.002*), none of the associations shown in Table 5 remained statistically significant.

**Table 5:** Correlations between SIRT1 and NAMPT and metabolic variables

	BMI	Weight	Waist	Triglycerides	Glucose	HbA1c
SIRT1 EAT	r = − 0.169	r = − 0.063	r = 0.023	r = − 0.238	r = − 0.091	**r = − 0.371**
	*p* = *0.023*	*p* = *0.66*	*p* = *0.87*	*p* = *0.095*	*p* = *0.055*	***p* = *0.012***
SIRT1 PAT	r = 0.005	r = 0.033	r = 0.083	r = -0.259	r = 0.006	r = -0.233
	*p* = *0.97*	*p* = *0.82*	*p* = *0.58*	*p* = *0.069*	*p* = *0.97*	*p* = *0.12*
SIRT1SAT	r = − 0.030	r = 0.091	r = − 0.010	**r = − 0.327**	r = − 0.004	r = − 0.225
	*p* = *0.84*	*p* = *0.52*	*p* = *0.95*	***p* = *0.022***	*p* = *0.98*	*p* = *0.14*
SIRT1 ng/mL	r = 0.222	**r = 0.384**	**r = 0.324**	r = − 0.130	r = − 0.031	**r = − 0.350**
	*p* = *0.11*	***p* = *0.005***	***p* = *0.026***	*p* = *0.37*	*p* = *0.84*	***p* = *0.018***
NAMPT EAT	r = − 0.151	r = − 0.063	r = 0.134	r = − 0.255	r = − 0.054	r = − 0.168
	*p* = *0.29*	*p* = *0.66*	*p* = *0.37*	*p* = *0.074*	*p* = *0.72*	*p* = *0.27*
NAMPT PAT	r = − 0.004	r = − 0.019	r = 0.107	r = − 0.012	r = 0.253	r = − 0.098
	*p* = *0.98*	*p* = *0.89*	*p* = *0.48*	*p* = *0.93*	*p* = *0.093*	*p* = *0.52*
NAMPT SAT	r = 0.097	r = 0.111	r = − 0.018	r = − 0.149	r = 0.249	r = − 0.177
	*p* = *0.50*	*p* = *0.44*	*p* = *0.91*	*p* = *0.31*	*p* = *0.11*	*p* = *0.45*
NAMPT ng/mL	r = 0.048	r = 0.163	r = 0.071	r = -0.144	r = 0.205	**r = 0.386**
	*p* = *0.74*	*p* = *0.26*	*p* = *0.64*	*p* = *0.33*	*p* = *0.19*	***p* = *0.010***

Bold text refers to statistically significant correlations before correcting for multiple testing

## Discussion

### The main result in the present study was the differently expressed SIRT1 and

NAMPT in EAT, PAT and SAT in CHD patients, with higher appearance in SAT compared to EAT of both markers. The whole first sentence should be the heading. The EAT and PAT expressions of both were reflected in SAT, NAMPT to a lesser extent, and with the lowest mirrored values between EAT and PAT of SIRT1. SIRT1 and NAMPT expressions were significantly inter-correlated in all three AT compartments, whereas their protein concentration were inversely associated in the circulation. SIRT1 was inversely and NAMPT positively correlated with the expression of pro-inflammatory cytokines, and they both inter-correlated with the Mɸ1 marker NOS2, SIRT1 possibly through compensatory mechanisms. Levels of of SIRT1 and NAMPT were similar in CHD patients and controls, which can be explained by their cardiopathy and an eventual pro-inflammatory state in the controls with aortic valvular disease [29].

The potential role of SIRT1 and NAMPT in AT remodeling in a population of overweight subjects with CHD will be dicussed in the following sections.

### The potential impact of SIRT1 in EAT, PAT and SAT

The impact of SIRT1 in the cardiovascular system seems to relate to its anti-inflammatory (inhibition of nuclear factor kappa-light-chain-enhancer of activated B cells (NF-_Κ_B)), anti-oxidative (dampened production of radical oxygen species (ROS)), and anti-apoptotic properties (deacetylation of p53 and class O of forkhead box transcription factors (FOXO) proteins) [11, 30]. Whether SIRT1s’ pleiotropic functions also are present in AT is likely, but not well known. Low SIRT1 expression in SAT of subjects with obesity has been shown [31] and circulating SIRT1 was reported to be inversely correlated to epicardial fat thickness in patients with obesity [14]. We observed that SIRT1 expression in SAT and PAT was similar, and higher than the expression in EAT. In our cohort with CHD, an ongoing accelerated inflammation is likely, mediated via several ways, such as the NF-_Κ_B and the NLRP3 inflammasome-related pathways. As the pro-inflammatory cytokine IL-18 is a downstream product of both pathways [32, 33] (the NLRP3 inflammasome also capable of activating NF-_Κ_B directly [34]), SIRT1 expression might have been downregulated in EAT, with respect to the observed lowest expression in EAT and the strong and inverse correlation between SIRT1 and IL-18 in EAT in our CHD population, and the previously reported significantly elevated IL-18 expression levels in EAT compared to PAT and SAT in the same cohort [5]. Considering this, the inflammatory status in EAT may reflect the current and progressive atherosclerotic process also taking place in the coronary arteries. At this point, with substantiated CHD in need of CABG, the pro-inflammatory macrophages (M1mɸ) are probably dominating in EAT and in the coronary arteries, but also in PAT and SAT, in which SIRT1 is upregulated, possibly by compensatory mechanisms in other cell types i.e., endothelial cells and probably also adipocytes, in an attempt of dampening the inflammation. This might be reflected in the observed positive correlation between SIRT1 and NOS2 in the different AT compartments, and as AT is vascularized, also the significant correlation between SIRT1 and the endothelial markers CD31 in SAT and PAT. NOS2 has recently been shown upregulated in EAT and PAT in these patients with LDL levels above the median value (1.8 mmol/L) [28]. The elevated SIRT1 expression in both SAT and PAT compared to EAT may be a consequence of the previously reported lower expressed IL-18 in these compartments compared to EAT [5]. We suggest that the SIRT1 profile has been prejudiced by a long-lasting chronic pro-inflammatory process in our CHD patients, with EAT being the most inflamed tissue compared to PAT and SAT. Based on our results, targeting SIRT in AT may be potential anti-inflammatory therapy in the heart, suggesting use nanocarriers i.e., liposomes due to nature of the adipose tissue, to deliver SIRT1 activating compounds *on-site* such as resveratrol or thiazole-based activators [35–37].

### The potential impact of NAMPT in EAT, PAT and SAT

NAMPT was similarly expressed in PAT and SAT. Its expression was significantly correlated to NLRP3, especially in PAT, and to the down-stream pro-inflammatory cytokine IL-1β, especially in SAT. NAMPT can activate the NLRP3 inflammasome [38], especially in obese, and NAMPT was reported to cause endothelial injury and inter-endothelial junction disruption in cultured mouse vascular endothelial cells [39]. Our cohort was not obese, but overweight, thus a plausible explanation of the observed results. It may be suggested, also based on other reports, that NAMPT might be an early and active player in the pro-inflammatory process starting in PAT and SAT, then transferring to EAT through paracrine signalling. NAMPT has been reported to be expressed in periadventitial and apical epicardial AT [40], and this local production has been suggested to play a role in myocardial fibrosis and remodelling [41] and in reverse left ventricular remodelling after aortic valve replacement [42]. With the observed correlation between NAMPT and NOS2, especially in SAT, although not significant after correcting for multiple testing, we may suggest that these infiltrating immune cells, especially M1mɸ (referring to the positive correlation to CD68 in PAT) have initiated or amplified NAMPT expression in AT.

### SIRT1 and NAMPT inter-relationship

SIRT1 is regulated by NAD+ bioavailability, partly mediated via NAMPT, although NAD+ can also be produced from tryptophan and nicotinic acid [43]. It has been demonstrated in mice, that deacetylation of intracellular NAMPT by SIRT1 predisposes the protein to secretion in adipocytes [44]. The compensatory upregulation of SIRT1 in endothelial cells and potentially also adipocytes may have contributed to elevated extracellular NAMPT, and its pro-inflammatory cytokine trait might have amplified the inflammation. The strong correlations between SIRT1 and NAMPT, especially in EAT and PAT, may thus support this assumption. One may have expected that with elevated levels of NAMPT, NAD+ would be elevated and indirectly increase the activity of SIRT1. However, NAD+ is a coenzyme in other systems, such as the nuclear enzymes poly (ADP-ribose) polymerases (PARPs) involved in cell repair, and CD38, a surface glycoprotein on immune cells. An increase in ROS leads to chronic PARP activation with rapid consumption of NAD+ , and consequently decreased SIRT activity [45]. Although speculatively, it might be suggested that with an ongoing chronic low-grade inflammation, available NAD+ has preferentially been consumed in these enzyme systems rather than by SIRT1.

As we observed that circulating levels of SIRT1 and NAMPT were not correlated to their expression in the different AT compartments, the main sources to levels in the circulation are probably not the AT. The inverse correlation between circulating SIRT1 and NAMPT, although weak, may point towards opposite systemic inflammatory mechanisms.

### SIRT1, NAMPT and cardiometabolic markers

SIRT1 expression was inversely associated with levels of triglycerides and HbA1c, as also previously reported by us and others [46, 47]. The possible chronic metabolic stress in the patients, some also with diabetes, may have implied reduced expression of SIRT1. As SIRT1 seems to be able to increase insulin sensitivity [48], dampened intracellular SIRT activity may have amplified an ongoing resistance to insulin. The raise in circulating SIRT1 levels along with increasing weight, also previously observed by our group in a healthy population [49], is suggested to be compensatory. It is important to notice that elevated expression and circulating levels of SIRT1 not necessarily reflect increased activity of the enzyme.

The correlation between NAMPT concentration and HbA1c, may be discussed to be related to glucose dysregulation. Although previously reported as a mediator in lowering glucose by activating the insulin receptor [50], these results were retracted [51], and the role of NAMPT in this context is still a controverse [41].

About 25% of the patients suffered from diabetes, which might have influenced the SIRT1 and NAMPT results, pointing towards an unhealthy metabolic state in the patients, despite treated with anti-diabetic and lipid-lowering drugs.

### Limitations

Despite achieved statistically significant results, the numbers are limited comprising statistical error Type II. As mRNAs are subject to regulation via different mechanisms such as epigenetic regulatory mechanisms, our results cannot verify abundance and/or activity of the proteins. Volume measurements of especially EAT and PAT would also have improved the results. The cohort consisted of mainly men (77%), which make the generalizability of results to women challenging. As no causality can be proved from the present work, the observed associations merely generate hypotheses which need to be further explored. The strength of the study is its explorative design with biopsies taken from EAT and PAT and SAT during open chest surgery.

## Conclusion

The gene expression of SIRT1 and NAMPT in EAT and PAT was partly reflected in SAT. Our results point towards a compensatory anti-inflammatory function of SIRT1 in AT, whereas NAMPT seems to function as a pro-inflammatory early player in SAT and PAT. With an unbeneficial remodelled AT, potentially also increased in size, the two investigated markers might opposingly have contributed to the inflammatory state. Targeting SIRT1 activity in the AT as potential therapy, using nanocarriers with SIRT1- activating compounds, to dampen inflammation in patients exposed to increased risk of CHD, might be a promising research field.

### Supplementary Information

**Additional file 1: Table S1.** Correlations of SIRT1 and NAMPT to NLRP3 inflammasome-related markers. **Table S2.** Correlations of SIRT1 and NAMPT to macrophage polarization markers

## Data Availability

The datasets used and analyzed during the current study are available from the corresponding author on reasonable request.

## Abbreviations

AT: Adipose tissue
CD: Cluster of differentiation
CHD: Coronary heart disease
EAT: Epicardial adipose tissue
FOXO: Class O of forkhead box transcription factors
HbA1c: Glycated haemoglobin
IL: Interleukin
NAD: Nicotinamide adenine dinucleotide
NAMPT: Nicotinamide phosphoribosyl transferase
NF-_Κ_B: Nuclear factor kappa-light-chain-enhancer of activated B cells
NLRP3: Nod-like receptor family pyrin domain containing 3
NOS2: Nitric oxide synthase 2
PAT: Pericardial adipose tissue
PCR: Polymerase chain reaction
PPAR: Poly (ADP-ribose) polymerase
ROS: Radical oxygen species
SAT: Subcutaneous adipose tissue
SIRT: Sirtuin1
