# Supplementary material for: Sirtuin1, not NAMPT, possesses anti-inflammatory effects in epicardial, pericardial and subcutaneous adipose tissue in patients with CHD
Source: J Transl Med. 2023 Sep 21;21:644. doi: 10.1186/s12967-023-04518-4 (PMC10512577; doi:10.1186/s12967-023-04518-4)
Supplement: Supplementary file 1 — Additional file 1: Table S1. Correlations of SIRT1 and NAMPT to NLRP3 inflammasome-related markers. Table S2. Correlations of SIRT1 and NAMPT to macrophage polarization markers [file 12967_2023_4518_MOESM1_ESM.docx]

**Additional files**

**Additional Table 1**

Correlations of SIRT1 and NAMPT to NLRP3 inflammasome-related markers

**Additional Table 2**

Correlations of SIRT1 and NAMPT to macrophage polarization markers

**Additional Table 1 Correlations of SIRT1 and NAMPT to the NLRP3 inflammasome-related markers in different AT**

**compartments.**

|  | IL-6  EAT | IL-6 PAT | IL-6  SAT | IL-6 R  EAT | IL-6 R PAT | IL-6 R  SAT | IL-18  EAT | IL-18  PAT | IL-18  SAT | IL-1β  EAT | IL-1β  PAT | IL-1β  SAT | NLRP3  EAT | NLRP3  PAT | NLRP3  SAT |
| --- | --- | --- | --- | --- | --- | --- | --- | --- | --- | --- | --- | --- | --- | --- | --- |
| SIRT1 EAT | r=-0.007  *p=0.96* | r=-0.073  *p=0.61* | r=-0.037  *p=0.80* | r=-0.181  *p=0.20* | r=0.053  *p=0.71* | r=-0.050  *p=0.73* | **r=-0.427***  ***p=0.002*** | r=-0.160  *p=0.26* | **r=-0.349**  ***p=0.014*** | r=0.031  *p=0.83* | r=-0.017  *p=0.91* | r=-0.135  *p=0.35* | **r=0.286**  ***p=0.039*** | r=0.056  *p=0.69* | r=0.041  *p=0.78* |
| SIRT1 PAT | r=-0.096  *p=0.50* | r=-0.002  *p=0.99* | r=-0.015  *p=0.92* | r=-0.080  *p=0.85* | r=0.123  *p=0.39* | **r=0.313**  ***p=0.029*** | r=-0.032  *p=0.82* | r=-0.074  *p=0.60* | r=-0.090  *p=0.54* | r=-0.092  *p=0.52* | r=-0.161  *p=0.25* | r=0.155  *p=0.29* | r=-0.146  *p=0.30* | r=0.247  *p=0.080* | r=0.156  *p=0.29* |
| SIRT1 SAT | r=0.000  *p=0.99* | r=0.005  *p=0.97* | r=0.015  *p=0.92* | r=0.157  *p=0.27* | r=0.052  *p=0.72* | **r=0.331**  ***p=0.020*** | r=0.106  *p=0.47* | r=-0.066  *p=0.64* | **r=-0.378**  ***p=0.007*** | r=0.119  *p=0.41* | r=0.113  *p=0.43* | r=0.017  *p=0.91* | r=-0.031  *p=0.83* | r=0.65  *p=0.65* | r=0.153  *p=0.30* |
| NAMPT  EAT | **r=0.367**  ***p=0.008*** | r=-0.110  *p=0.44* | r=-0.070  *p=0.64* | r=-0.202  *p=0.15* | r=-0.025  *p=0.86* | r=-0.065  *p=0.66* | **r=-0.350**  ***p=0.012*** | r=0.067  *p=0.64* | r=0.050  *p=0.73* | r=0.196  *p=0.16* | r=-0.065  *p=0.65* | r=-0.056  *p=0.70* | **r=0.383**  ***p=0.005*** | r=0.124  *p=0.39* | r=-0.057  *p=0.70* |
| NAMPT  PAT | **r=0.296**  ***p=0.035*** | r=0.241  *p=0.089* | r=0.099  *p=0.50* | r=-0.033  *p=0.82* | **r=0.283**  ***p=0.044*** | r=0.188  *p=0.197* | r=0.113  *p=0.43* | r=-0.055  *p=0.70* | r=-0.068  *p=0.64* | r=-0.168  *p=0.24* | r=0.112  *p=0.43* | r=0.145  *p=0.32* | r=-0.042  *p=0.77* | **r=0.456***  ***p=0.001*** | r=0.021  *p=0.88* |
| NAMPT  SAT | r=-0.196  *p=0.18* | r=0.091  *p=0.53* | **r=0.371**  ***p=0.009*** | r=-0.090  *p=0.54* | r=0.048  *p=0.74* | **r=0.353**  ***p=0.014*** | r=0.179  *p=0.22* | r=0.126  *p=0.38* | r=-0.064  *p=0.67* | r=-0.243  *p=0.089* | r=0.188  *p=0.19* | **r=0.548***  ***p<0.001*** | **r=-0.305**  ***p=0.031*** | r=0.100  *p=0.50* | **r=0.377**  ***p=0.018*** |

Bold text refers to statistically significant correlations (*p<0.05*)

* Refers to statistically significant correlations after Bonferroni correction (illustrated in Figure 2)

**Additional Table 2 Correlations of SIRT1 and NAMPT to the macrophage polarizations markers NOS2 (Mɸ1) and CD206 (Mɸ2) in different AT compartments.**

|  | CD206 EAT | CD206 PAT | CD2 206 SAT | NOS2 EAT | NOS2 PAT | NOS2 SAT |
| --- | --- | --- | --- | --- | --- | --- |
| SIRT1 EAT | r=0.180  *p=0.20* | r=-0.149  *p=0.29* | r=-0.053  *p=0.71* | r=0.201  *p=0.26* | r=0.167  *p=0.37* | r=0.122  *p=0.54* |
| SIRT1 PAT | r=0.047  *p=0.74* | r=0.257  *p=0.066* | r=0.155  *p=0.42* | **r=0.550***  ***p=0.001*** | **r=0.448**  ***p=0.011*** | **r=0.451**  ***p=0.018*** |
| SIRT1 SAT | r=-0.069  *p=0.63* | r=-0.240  *p=0.090* | r=0.162  *p=0.26* | **r=0.355**  ***p=0.042*** | **r=0.373**  ***p=0.043*** | **r=0.518***  ***p=0.006*** |
| NAMPT EAT | **r=0.306**  ***p=0.027*** | r=-0.113  *p=0.42* | r=-0.114  *p=0.42* | r=0.234  *p=0.18* | r=0.193  *p=0.30* | r=0.015  *p=0.94* |
| NAMPT PAT | r=-0.154  *p=0.27* | r=0.257  *p=0.066* | r=-0.052  *p=0.72* | **r=0.370**  ***p=0.031*** | r=0.339  *p=0.062* | r=0.153  *p=0.45* |
| NAMPT SAT | r=-0.077  *p=0.59* | r=-0.017  *p=0.90* | r=-0.050  *p=0.73* | r=0.277  *p=0.13* | r=0.152  *p=0.43* | **r=0.497**  ***p=0.010*** |

Bold text refers to statistically significant correlations (*p<0.05*)

* Refers to statistically significant correlations after Bonferroni correction (illustrated in Figure 3)
